# Annotations

**Published:** 1901-10-05

**Authors:** 


### ANNOTATIONS.

### The Conscience Clause in the Vaccination Act.

Under the stress of the presence of smallpox a good many parents are just now being brought before the magistrates for non-compliance with the Vaccination Act, which, so far as it goes, is satisfactory. A good many applications are also being made for certificates of exemption under what is called the conscience clause, and the manner in which these applications are being dealt with by the magistrates is not, on the other hand, all that might be wished. An applicant, for example, says: "I will not have my child vaccinated," and the magistrate very kindly argues the matter with him. The father, however, sticks to his point, says that he understands the responsibility and is prepared to take the risk, and on this he gets his certificate. But there is nothing in the Act to say that a man who refuses to have his child vaccinated, or who understands the risk and is willing to take it, is to be let off. The Act merely says that no penalty shall lie if within four months of the birth of the child the parent satisfies the magistrate "that he conscientiously believes that vaccination would be prejudicial to the health of the child," and delivers a certificate to that effect, signed by the magistrate, to the vaccination officer. If a man merely says that he objects, or that he will take the risk, or makes any excuse of like nature, all that magistrate need do is to dismiss the application without further parley. It is only when the applicant pleads the particular objection mentioned in the Act that the magistrate is in any way bound to intervene, and there can be but little doubt that a considerable number of the cases in which certificates have been granted of late would have been dismissed if without any discussion the magistrates had merely refused to listen to excuses outside the strict wording of the Act.

### Chill as a Cause of Disease.

Some remarks recently made by Professor Clifford Allbutt in regard to the influence of chill as a cause of disease serve to illustrate how quickly the pendulum of medical fashion swings as new ideas gain currency in the minds of medical practitioners. Time was when all sorts of ailments were with the utmost confidence attributed to "chill," and when physicians in gravely putting down the origin of colds, pneumonias, and fevers of all kinds to this cause, did so without the slightest suspicion that they were talking nonsense. Then came the time when with one accord the profession bent the knee before the microbe. In proportion as we accepted the theory that febrile and even other maladies were due to germs and micro-organisms, it seemed absurd to talk of chill as provocative of such ailments. We laughed at the ignorance of our fathers, and if here and there a few of us, being sceptical, still talked of chill as an efficient cause of illness, we spoke with bated breath, for the microbe held the field. Then came the turn of the tide. It soon became evident that these wonderful germs were present in all sorts of unexpected places, and that if they really possessed the powers which some had attributed to them we ought all of us long ago to have been dead men. So we looked around for "predisposing" or contributory causes, influences by aid of which the microbes were enabled to take root, and among these we soon found our old friend "chill." As Professor Allbutt says, clinical physicians in their respect for bacteriology had of late rarely ventured to invoke chill and the like as causes of pneumonia, pleurisy, dysentery, and so forth; but, as things were now, he would urge that, so far from being remote, these might often be the immediate causes—so-called exciting causes; in other words, that of a score of persons carrying morbific bacteria about them only the few subjected to the immediate cause of chill might become affected with disease. So our grandmothers were right after all!

### The Boer Concentration Camps.

A Parliamentary paper (Cd. 789) was issued on September 26th showing the numbers and mortality in the camps of refuge in South Africa in August, 1901. In Natal, Cape Colony, Orange River Colony, and the Transvaal, the total number of whites was 105,347, and coloured 32,272. The death rate among the former was only 17·8, and the latter 14·4, in spite of the fact that a severe epidemic of measles was raging at the time. These figures compare favourably with similar ones relating to any of the large towns in England, and should do much to prove that many of the reports concerning the condition of these camps are gross exaggerations. The mortality is highest in the Transvaal and lowest in Natal, but considering that the season was winter, the management of the camps must have been carried out very efficiently in order to show such low figures all round. Unfortunately the accounts from witnesses on the spot contradict each other absolutely, and have therefore little value as evidence, nor have we any reliable data on which to base a comparison between the death rate in the camps, and that in times of peace among the Boers, but we may safely assume that it would be but little, if at all, less than at present in the camps. That the concentration of so many is favourable to the spread of epidemics cannot be denied, but, on the other hand, the people have the opportunity of learning some of the elements of sanitation, and the necessity of cleanliness, and with good wholesome food, should not suffer from disease more than, if as much, as the ordinary being who is compelled to spend his life in some crowded town.

### Reservoirs and Rivers.

Those who have maintained through thick and thin the virtues of the Thames as a source from which to draw water for the supply of London will take comfort and much encouragement from the reports of water famines in the north of England, where some of the towns which depend upon reservoirs alone are suffering dire distress. It is possible, and indeed it seems not improbable, that London will before long have to obtain access to some new source of supply. The advantages, however, of having access to the immense natural reservoir provided by the water bearing country drained by the Thames, in addition to any artificial system which we construct, are so great that we may be pretty sure that, with the knowledge of the risks run by exclusively reservoir-fed towns, London will not be easily tempted to desert its old friend the River Thames, and it is to be hoped that nothing will tempt the Thames Conservators to slacken their efforts to maintain the purity of the river which forms London's great natural source of supply.

